# Analysis of an RNA-seq Strand-Specific Library Sample Reveals a Complete Genome of *Hardenbergia mosaic virus* from Native Wisteria, an Indigenous Virus from Southwest Australia

**DOI:** 10.1128/genomeA.00599-17

**Published:** 2017-07-27

**Authors:** Solomon Maina, Roger A. C. Jones

**Affiliations:** a School of Agriculture and Environment, Faculty of Science, University of Western Australia, Crawley, Western Australia, Australia; b Institute of Agriculture, Faculty of Science, University of Western Australia, Crawley, Western Australia, Australia; c Department of Agriculture and Food Western Australia, South Perth, Western Australia, Australia

## Abstract

Analysis of an RNA-seq strand-specific library revealed a complete genome of *Hardenbergia mosaic virus* (HarMV) from RNA extracted from a native wisteria (*Hardenbergia comptoniana*) plant from southwest Australia. We compared it with eight other complete HarMV genomes. It most resembled (85.8% nucleotide identity) the genome of HarMV isolate MD4-D.

## GENOME ANNOUNCEMENT

In July 2016, an apical leaf sample showing obvious mosaic and deformation symptoms was collected from a plant of the indigenous southwest Australian species native wisteria (*Hardenbergia comptoniana*; family *Fabaceae*). The plant sampled also exhibited symptoms typical of phytoplasma infection (leaf chlorosis, proliferation of axillary shoots, and stunting). It was growing on a fence line bordering a playing field area in the Victoria Park suburb of Perth in southwest Australia. *Hardenbergia mosaic virus* (HarMV; genus *Potyvirus*, family *Potyviridae*) causes a conspicuous disease in native wisteria plants, is spread nonpersistently by aphids, and, like native wisteria itself, is indigenous to the region (1–5). It invades introduced lupin species (*Lupinus* spp.) at the interface of the local ancient ecosystem and recent agroecosystem (6, 7). Analysis of polyadenylated transcripts derived from RNAseq-stranded libraries (8–17) prepared from RNA extracted from the collected sample (designated VPK) detected one complete HarMV genome.

RNA was extracted from the VPK sample using a ZR Plant RNA MiniPrepTM kit (Zymo Research) and treated with RNase-free DNase (Invitrogen). The extract was subjected to library preparation using a TruSeq-stranded Ribo-Zero plant kit (Illumina, catalog no. RS-122-2401) and was subsequently subjected to quality control (8–17). The library was sent to Macrogen, Inc. (South Korea), where sequencing was done using the HiSeq 2500 platform with a TruSeq SBS version 4 kit (Illumina) with 151 cycles of paired-end reads. Reads were then assembled and genomes annotated using CLC Genomics Workbench version 6.5 (CLC bio) and Geneious version 8.1.7 (Biomatters) (18, 19).

The VPK sample yielded 13,609,056 reads and, after trimming, 12,972,958 remained. *De novo* assembly generated 25 contigs and 622,179 reads mapped to the contig of interest with a coverage of 9,107×. The complete genome obtained was named VPK-1. It consisted of 9,621 nucleotides (nt) and coded for 10 proteins, which is similar to other potyviruses (20, 21). There were eight other complete HarMV genomes already in GenBank (3, 7). A BLAST-based search (22) revealed that sequence VPK-1 most resembled the sequence of HarMV isolate MD4-D (KJ152157) with an 85.8% nt identity. In addition, the analysis revealed a partial sequence (6,002 nt in length) named VPK-2, which belonged to a different HarMV strain. When sequences VPK-1 and MD4-D were truncated to match partial sequence VPK-2, the VPK-2 sequence was only distantly related to VPK-1 (83.1% nt identity) and MD4-D (82.5% nt identity). This study provides yet another example of the accuracy and reliability of high-throughput sequencing using both *de novo* and reference assembly approaches to separate different strains of the same virus present in the same sample (3, 7, 23, 24). There was no agriculture in southwest Australia until European colonization in 1829 but the region has a very diverse native flora. This diversity is reflected by the wide genetic diversity found within HarMV in this region (1–7). Such diversity is typical of indigenous viruses that have evolved over a very long period in isolation in native vegetation in remote regions (1–5, 25).

### Accession number(s).

Sequence VPK-1 has been deposited in GenBank under the accession number MF040762.
